# Somatic Embryogenesis and Massive Shoot Regeneration from Immature Embryo Explants of Tef

**DOI:** 10.4061/2011/309731

**Published:** 2011-10-18

**Authors:** Likyelesh Gugsa, Jochen Kumlehn

**Affiliations:** ^1^Plant Reproductive Biology, Department of Physiology and Cell Biology, Leibniz Institute of Plant Genetics and Crop Plant Research (IPK) Gatersleben, Corrensstraße 3, 06466 Gatersleben, Germany; ^2^Ethiopian Institute of Agricultural Research (EIAR) Holetta, P.O. Box 2003, Addis Ababa, Ethiopia

## Abstract

Tef (*Eragrostis tef*) provides a major source of human nutrition in the Horn of Africa, but biotechnology has had little impact on its improvement to date. Here, we report the elaboration of an *in vitro* regeneration protocol, based on the use of immature zygotic embryos as explant. Explant size was an important determinant of *in vitro* regeneration efficiency, as was the formulation of the culture medium. Optimal results were obtained by culturing 0.2–0.35 mm embryo explants on a medium containing KBP minerals, 9.2–13.8 *μ*M 2,4-dichlorophenoxyacetic acid, 6 mM glutamine, and 0.5% Phytagel. Although this protocol was effective for both the improved cultivar “DZ-01-196” and the landrace “Fesho”, the former produced consistently more embryogenic tissue and a higher number of regenerants. An average of more than 2,800 shoots could be obtained from each “DZ-01-196” explant after 12 weeks of *in vitro* culture. These shoots readily formed roots, and plantlets transferred to soil were able to develop into morphologically normal, fertile plants. This regeneration and multiplication system should allow for the application of a range of biotechnological methods to tef.

## 1. Introduction

The small-grained cereal tef [*Eragrostis tef (*Zucc.) Trotter] is indigenous to Ethiopia, which is its centre of diversity and site of domestication [1]. The species is an allotetraploid (2*n* = 4*x* = 40), facultatively self-pollinating C_4_-plant [2, 3], and its gluten-free grain contains a high content of bioavailable iron, essential amino acids, and complex carbohydrates. While the crop is well adapted to a range of climatic and soil conditions and enjoys a good level of tolerance to many pests and diseases, its mean grain yield in cultivation lies below 1t ha^−1^ [4]. Nevertheless, the crop is cultivated over about one-third of the arable area in Ethiopia and represents a staple food for much of the population, in the form of the traditional pancake *injera*. Lodging is a major barrier to increasing productivity and has yet to be overcome by conventional breeding approaches or interspecific hybridization, so probably other strategies, such as mutagenesis or genetic transformation, are needed. To date, little investment in biotechnology has been applied to tef. *In vitro* plant regeneration from explants such as roots, young leaf bases, or seeds has been demonstrated [5–7], but the levels of efficiency achieved remain too low for use in tef improvement. 

A viable method of doubled-haploid formation using *in vitro* gynogenesis has been described by Gugsa et al. [8], but what remains missing for effective embryo rescue and genetic transformation in tef is a means to induce somatic embryogenesis from which multiple *de novo *shoots can be regenerated. The very small size of the tef caryopsis (1.0–1.6 mm) means that the excision of the immature embryos is technically challenging [7]—this explant type is widely used to generate somatic embryos in other cereal species [9]. 

Here, we detail the elaboration of an *in vitro *culture system for immature tef embryos which has the capacity to differentiate somatic embryos able to form multiple shoots and viable regenerant whole plants. 

## 2. Materials and Methods

### 2.1. Plant Materials and Surface Sterilization

**Figure 1:**
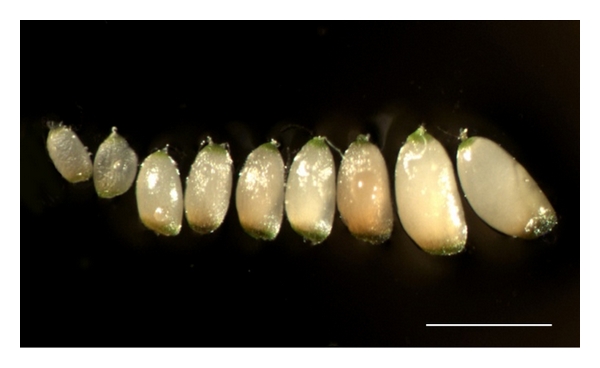
Developmental stages of tef caryopses used to excise immature embryos. Bar = 1 mm.

Grain of the tef land race “Fesho” and the widely grown improved variety “DZ-01-196” (cv. “Magna”) were germinated in peat-soil mix in a glasshouse at ~24°C. Plantosan fertilizer (Aglucon, Germany) was applied at 12 g per 16 L pot three weeks after planting. Around 7–10 d after the onset of anthesis, panicles were cut at the peduncle and each placed in a 50 ml screw-capped plastic centrifuge tube to allow surface sterilization of the immature caryopses by shaking for 15 min in 1%  w/v sodium hypochlorite, followed by rinsing 3-4 times in sterile doubled distilled water. The immature embryos were recovered from the detached caryopses (Figure 1) by squeezing them out through an incision made at the base of the caryopsis. 

### 2.2. Callus and Somatic Embryo Formation

**Figure 2:**
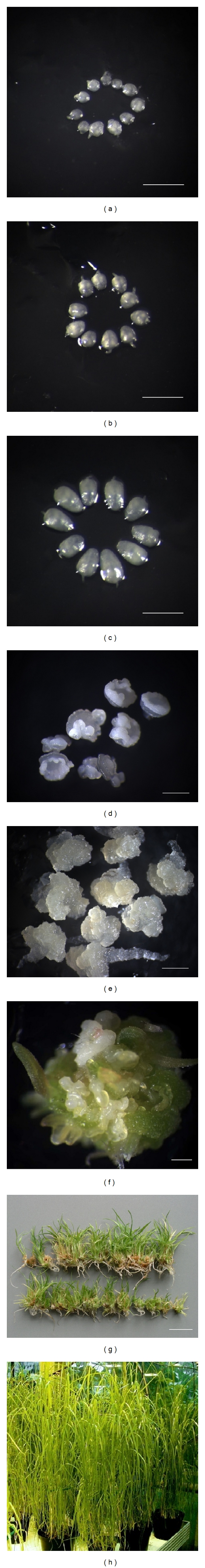
Regeneration of tef plants from immature embryo explants via somatic embryogenesis. Three-size classes of immature embryos were cultivated: small (a, 0.1–0.2 mm, bar = 0.5 mm), intermediate-sized (b, 0.2–0.35 mm, bar = 0.5 mm), and large (c, 0.35–0.75 mm, bar = 0.5 mm). After two weeks of culture on callus induction medium, small explants of cv. “DZ-01-196” typically showed direct somatic embryogenesis with no callus formation being detectable (d, bar = 0.5 mm), whereas large explants first generated callus, which later differentiated somatic embryos (e, bar = 1 mm). Upon transfer of explants to regeneration medium and exposure to light, somatic embryos further developed and shoots appeared within two weeks (f, bar = 1 mm). A high number of shoots can be obtained from one of the callus fragments subcultured, especially when intermediate-sized zygotic embryos are used (g, bar = 10 mm). Upon root formation on K4NB medium, plantlets were transferred to soil, where they developed normally.

Small (0.1–0.2 mm), intermediate-sized (0.2–0.35 mm), and large (0.35–0.75 mm) embryos were sorted on the basis of size and plated scutellum side up at 7–10 embryos per 3.5-cm Petri dish (Figures 2(a)–2(c)) containing 3 mL callus induction medium, which were sealed with Nescofilm (Carl Roth GmbH, Germany) and incubated in the dark at 25 ± 2°C. A comparison was first made between callus induction media formulations comprising the mineral salts present in MS [10], N6 [11], KBP [12], and L3 [13] media, to which were added the organic supplements specified by Kao and Michayluk [14] in the form of a commercially available product (K3129, Sigma-Aldrich, USA), 3 mM glutamine, 10 mM morpholinoethanesulfonic acid (MES), 75 *μ*M NaFeEDTA, 250 mM maltose (as sole carbohydrate source), 9.2 *μ*M 2,4-dichlorophenoxyacetic acid (2,4-D), pH 5.8, and solidified with 0.4% Phytagel (Sigma-Aldrich, USA), if not stated otherwise. Except for the Phytagel, which was autoclaved as 1.5% stock, the above components were all filter-sterilized before preparation of the media. The small and intermediate-sized “DZ-01-196” embryos required two weeks of culture to form somatic embryos, while the large ones required three weeks. The “Fesho” embryos, irrespective of their size, needed to be transferred to fresh callus induction medium after two weeks and held for a further two weeks, because somatic embryos emerged later than from “DZ-01-196” explants. Comparative experiments were also conducted to explore the benefit of varying the concentration of 2,4-D (4.6, 9.2, 13.8, 18.4 *μ*M), glutamine (0, 1, 3, 6, 10 mM), and Phytagel (0.2, 0.3, 0.4, 0.5, 0.6%), as well as determining the effect of embryo size. 

### 2.3. Shoot Formation and Plant Regeneration

Explants which developed callus and/or somatic embryos (also referred to as *responding embryos*) were transferred to 9 cm Petri plates containing 15 mL antibiotic-free K4NB regeneration medium [12] and incubated for eight weeks at 24 ± 2°C, with 16 h per day artificial light provided by cool white florescent tube lights. Subculturing was carried out every two weeks.

### 2.4. Validation of the Optimized Protocol

In a final experiment, “DZ-01-196” explants of each embryo size class were fragmented into 5–10 pieces after two successive sub-cultures of two weeks each on optimized callus induction medium (KBP minerals, 6 mM glutamine, 0.5% Phytagel, all other components as above) and transferred to K4NB regeneration medium, with subcultivation and further fragmentation being conducted fortnightly over a period of eight weeks. Where necessary, nonrooted shoots were cultivated on the same medium to induce root formation. Rooted plantlets were potted in soil and grown in a greenhouse, as described above for the donor plants. 

### 2.5. Statistical Analysis

Three independent replicates were set up in experiments designed to optimize the callus induction medium, with each replicate consisting of two or three Petri dishes with 7 to 10 zygotic embryos each. For the validation of the optimized protocol, each replicate comprised four embryos per embryo size class. The software package SigmaStat v3.0 (SPSS Inc., USA) was used for all statistical calculations. The data were interpreted either by conventional ANOVA, or—where the data failed either a normality or an equal variance test—by the nonparametric Kruskal-Wallis test. Pairwise multiple comparisons of the different treatments were conducted using the Student-Newman-Keuls method. 

## 3. Results and Discussion

### 3.1. Effect of Mineral Nutrition during Callus and Somatic Embryo Formation

The large (0.35–0.75 mm) “Fesho” and “DZ-01-196” explants cultivated on media containing N6 and L3 minerals displayed a low level of somatic embryogenesis and very little, if any, differentiation of shoots. In contrast, both MS and KBP minerals were permissive of callus formation, somatic embryogenesis, and *de novo* shoot regeneration from both genetic stocks, with the latter medium giving significantly the best response (Table 1). KBP is also highly effective for generating embryogenic culture from barley pollen [12]. The MS medium is widely used for plant regeneration and genetic transformation systems, included in the cereals [15, 16] and has been shown to be effective for inducing embryogenesis and plant regeneration from unfertilized tef pistils [8].

### 3.2. Effect of 2,4-D during Callus and Somatic Embryo Formation

The initiation of callus appeared to be independent of both 2,4-D concentration and genotype, but somatic embryogenesis and in particular shoot formation were significantly influenced by the former. In intermediate-sized zygotic embryos, the medium range of 2,4-D concentration (9.2 and 13.8 *μ*M) was more effective than either the lowest or the highest concentrations tested (Table 2). A similar response was obtained using embryos of both the small and the large size classes of both stocks (data not shown), although the overall level of efficiency was less than for the intermediate-sized embryos. 

A level of 9.2 *μ*M 2,4-D has been reported to be optimal for the induction of regenerable callus from mature tef grains and leaf base segments [6, 7], while Gugsa et al. [8] have reported that 18.4 *μ*M 2,4-D was superior to 9.2 *μ*M for the formation of regenerable callus from unfertilized tef pistils. 

### 3.3. Effect of Glutamine during Callus and Somatic Embryo Formation

Supplementation of culture media with glutamine, a major plant nitrogen transport form, has been shown to enhance somatic embryogenesis, for instance in the gymnosperms [17] and cereals [18]. The glutamine concentration in the medium had a noticeable effect on the formation of immature somatic embryos in both “Fesho” and “DZ-01-196”, with the presence of 6 mM being associated with the highest proportion of intermediate-sized explants able to proceed to somatic embryogenesis and shoot differentiation (Table 3). The small and large explants behaved in a similar fashion, although the highest frequency of shoot formation from small “Fesho” explants was achieved in the presence of only 3 mM glutamine (data not shown). 

### 3.4. Effect of Zygotic Embryo Size

Intermediate-sized zygotic embryos of both “DZ-01-196” and “Fesho” produced significantly more shoots than did either the small or large ones. The *in vitro *growth of small and intermediate-sized “DZ-01-196” explants was noticeable after just three days, with somatic embryogenesis being initiated at the edges of the scutellum prior to callus formation (Figure 2(d)), a phenomenon called direct somatic embryogenesis. By contrast, the large explants tended to initially form callus before somatic embryos appeared on the callus surface during the third week of culture (Figure 2(e)). Direct somatic embryogenesis occurred only rarely from “Fesho” explants, and somatic embryogenesis required some two weeks longer and was less frequent than from “DZ-01-196” material. Somatic embryogenesis and the formation of well differentiated somatic embryos was more pronounced in cultured intermediate-sized and large embryos of both “DZ-01-196” and “Fesho”, especially after transfer of the explants to regeneration medium and exposure to light (Table 4). 

The influence on the efficacy of regenerable callus formation of the developmental stage reached by the immature embryo at the time of its excision has been demonstrated for a number of grass species. Thus, in both barley and wheat, a substantial loss in totipotency has been associated with the change in the explant's appearance from translucence to opacity [19]. In tef, the small and intermediate-sized explants were still translucent and were more likely to proceed to direct somatic embryogenesis than the larger starchy ones. Although the intermediate-sized explants proved to be the most efficient in terms of scutellum-derived somatic embryogenesis and shoot formation, the small ones also had a reasonable level of totipotency. However, their safe dissection is practically difficult, and if too small, they may not be capable of callus and somatic embryo formation without the provision of nurse tissue. Embryo size is of particular relevance to tef, given that they are in any case rather small compared to those from most cereal species. Unfortunately, *de novo* shoot formation from cultured mature caryopses, as reported by Assefa et al. [7], is rather slow and only few shoots can be obtained per explant. 

### 3.5. Effect of Medium Solidification during Callus and Somatic Embryo Formation

Plant cell cultures are conventionally grown on the surface of solidified media, not only to prevent submergence, but also to simplify the control of the water status of the regenerating tissue. The nature and concentration of the solidifying agent determines the ease with which water and nutrients can be accessed by the tissue. KBP-based media, supplemented with 6 mM glutamine, were solidified with various concentrations of Phytagel to compare their capacity to support the growth of regenerable tissue from “DZ-01-196” explants. Shoot formation from intermediate-sized explants was maximal in the presence of 0.5% Phytagel (Table 5). The small explants performed best on 0.3–0.5% Phytagel, while for the large ones, no statistically significant effect of Phytagel concentration was noted (data not shown). Irrespective of their size class, explants cultivated on 0.2% Phytagel tended to produce rapidly growing callus which seldom progressed to somatic embryogenesis. On 0.6% Phytagel, the explants grew somewhat slowly, but tended to achieve direct somatic embryogenesis rather than differentiating somatic embryos from callus tissue. 

### 3.6. Validation of an Optimized Protocol

**Figure 3:**
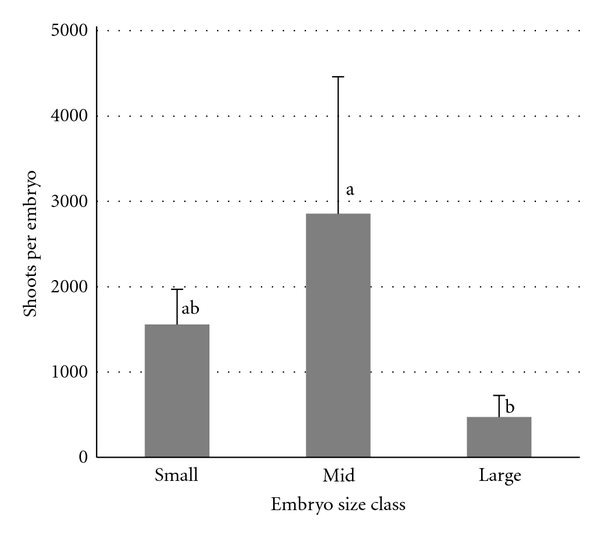
Multiple shoot formation from four “DZ-01-196” immature embryos per explant size class. After four weeks culture on callus induction medium, the calli were divided into fragments, transferred to regeneration medium for eight weeks, with sub-cultivation and further fragmentation imposed at fortnightly intervals. Mean numbers of shoots (represented by columns) associated with exclusively different letter superscript differ significantly from one another at *P* < 0.05.

The optimized conditions (callus induction medium containing KBP minerals, 6 mM glutamine, 9.2 *μ*M 2,4-D, and 0.5% Phytagel, followed by transfer to K4NB regeneration medium) were eventually validated by an experiment based on “DZ-01-196” explants. All the intermediate-sized explants regenerated multiple shoots. On the K4NB medium, not only did shoots and roots develop, but also proliferation continued and further somatic embryogenesis occurred (Figure 2(f)), resulting in the regeneration of many shoots from a single explant (Figure 2(g)). Through successive callus fragmentation and subculturing, a mean of >2,800 shoots per individual intermediate-sized zygotic embryo was obtained (Figure 3). All rootless shoots individually subcultivated on K4NB medium readily formed roots. A random sample of >100 plantlets was transferred to soil, and all developed into phenotypically normal and fertile plants (Figure 2(h)). 

## 4. Conclusions

We have demonstrated that immature tef embryos, even though they are rather small, are still amenable to *in vitro *culture and can be induced to form either callus and/or somatic embryos. The optimized protocol allows many regenerants to be rapidly produced from a single explant, with no sign of frequent morphological abnormalities. We believe that this protocol will find applications such as the rapid multiplication of valuable germplasm, embryo rescue in wide crosses, mutation breeding, haploid technology, and genetic transformation; a set of technologies which can now be brought to bear to overcome the major breeding bottlenecks affecting the productivity and end-use quality of tef.

## Figures and Tables

**Table 1:** The effect of the mineral nutrient composition of the callus induction medium on callus formation, somatic embryogenesis, and shoot regeneration of intermediate-sized and large “Fesho” and “DZ-01-196” immature zygotic embryos. Values (within one row) followed by different superscript letters are significantly different from one another at *P* < 0.05.

Genotype	Fesho				DZ-01-196			
Mineral nutrients	MS	N6	KBP	L3	MS	N6	KBP	L3
*No. of cultivated embryos*	60	60	60	60	60	60	60	60
% Responding embryos*	91.7^b^	53.3^c^	100^a^	33.3^d^	88.3^b^	66.7^c^	100^a^	48.3^d^
% Embryos forming somatic embryos	25.0^b^	6.7^c^	46.7^a^	1.7^c^	35.0^b^	18.3^c^	63.3^a^	2.9^d^
% Embryos regenerating shoots	18.3^b^	1.7^c^	33.3^a^	0^c^	21.7^b^	1.7^c^	48.3^a^	0^c^

*Embryos showing callus formation and/or somatic embryogenesis.

**Table 2:** The effect of 2,4-D concentration in the callus induction medium on callus formation, somatic embryogenesis, and shoot regeneration of intermediate-sized and large “Fesho” and “DZ-01-196” immature zygotic embryos. Values (within one row) followed by exclusively different superscript letters are significantly different from one another at *P* < 0.05.

Genotype	Fesho				DZ-01-196			
2,4-D [*μ*M]	4.6	9.2	13.8	18.4	4.6	9.2	13.8	18.4
*No. of cultivated embryos*	35	38	36	33	43	35	43	25
% Responding embryos*	54.3^a^	68.4^a^	83.3^a^	66.7^a^	88.4^a^	80.0^a^	86.0^a^	84.0^a^
% Embryos forming somatic embryos	22.9^b^	52.6^ab^	66.7^a^	12.1^c^	37.2^a^	74.3^a^	81.4^a^	44.0^a^
% Embryos regenerating shoots	11.4^b^	39.5^a^	47.2^a^	9.1^b^	20.9^c^	60.0^ab^	58.1^a^	8.0^b^

*Embryos showing callus formation and/or somatic embryogenesis.

**Table 3:** The effect of glutamine in the callus induction medium on callus formation, somatic embryogenesis, and shoot regeneration of intermediate-sized and large “Fesho” and “DZ-01-196” immature zygotic embryos. Values (within one row) followed by different superscript letters are significantly different from one another at *P* < 0.05.

Genotype	Fesho					DZ-01-196				
Glutamine [mM]	0	1	3	6	10	0	1	3	6	10
*No. of cultivated embryos*	75	75	75	75	75	60	60	62	69	60
% responding embryos*	46.7^c^	28.0^d^	57.3^b^	94.7^a^	90.7^a^	66.7^a^	38.3^b^	96.8^a^	100^a^	100^a^
% Embryos forming somatic embryos	14.7^d^	10.7^d^	56.0^b^	84.0^a^	40.0^c^	35.0^c^	25.0^d^	71.0^b^	94.2^a^	23.3^d^
% Embryos regenerating shoots	4.0^b^	4.0^b^	40.0^b^	68.0^a^	8.0^b^	31.7^c^	15.0^d^	66.1^b^	88.4^a^	0^e^

*Embryos showing callus formation and/or somatic embryogenesis.

**Table 4:** The effect of immature zygotic embryo size (small: 0.1–0.2 mm, mid: 0.2–0.35 mm, large: 0.35–0.75 mm) on callus formation, somatic embryogenesis, and shoot regeneration. Values (within one row) followed by different superscript letters are significantly different from one another at *P* < 0.05.

Genotype	Fesho			DZ-01-196		
Embryo size	small	mid	large	small	mid	large
*No. of cultivated embryos*	65	75	75	67	69	65
% Responding embryos*	100^a^	94.7^a^	78.7^b^	100^a^	100^a^	100^a^
% Embryos forming somatic embryos	76.9^a^	84.0^a^	65.3^b^	82.1^b^	94.2^a^	78.5^b^
% Embryos regenerating shoots	55.4^b^	68.0^a^	41.3^c^	62.7^c^	88.4^a^	69.2^b^

*Embryos showing callus formation and/or somatic embryogenesis.

**Table 5:** The effect of Phytagel concentration in the callus induction medium on the development of intermediate-sized “DZ-01-196” immature zygotic embryos. Values (within one row) followed by exclusively different superscript letters are significantly different from one another at *P* < 0.05.

Phytagel [%]	0.2	0.3	0.4	0.5	0.6
*No. of cultivated embryos*	46	65	67	78	78
% Responding embryos*	100^a^	90.8^a^	100^a^	100^a^	66.7^a^
% Embryos forming somatic embryos	50.6^b^	62.9^ab^	95.5^a^	100^a^	67.3^ab^
% Embryos regenerating shoots	20.0^b^	45.6^b^	82.2^a^	100^a^	42.1^b^

*Embryos showing callus formation and/or somatic embryogenesis.
